# Death of Dr. Rainey

**Published:** 1914-02

**Authors:** 


DEATH OF DR. RAINEY.—Dr. Frank Rainey, who was Superintendent of the Texas School for the Blind more than twenty-one years, died in Austin, February 2d (inst.), of arterio-sclerosis. He was widely known and greatly beloved by all who knew him; a genial and delightful companion and a wise physician. He was prominent in Masonic circles, and his funeral was conducted by the members of the local lodge. When he resigned some years ago from the position of Superintendent of the Blind School, he accepted that of Superintendent of the Masonic Home at Fort Worth. In recent years he was retired from all work.

Dr. Rainey was born in Green county, Alabama, in 1836; studied medicine with his brother-in-law, Dr. Merriwether, of Houston county; graduated M. D. from Tulane in 1858, and settled at Crockett, that county. He married Hulda Merriwether in 1860; entered the Confederate service in Green's command, and served till the close of the war. Peace to his ashes!

